# [Retracted] Pin2/TRF1‑binding protein X1 inhibits colorectal cancer cell migration and invasion in vitro and metastasis in vivo via the nuclear factor‑κB signaling pathway

**DOI:** 10.3892/or.2023.8477

**Published:** 2023-01-09

**Authors:** Tao Jiang, Haiqing Li, Ranran Jiang, Hailong Li, Pingfu Hou, Yansu Chen, Jin Bai, Jun Song

Oncol Rep 40: 1533–1544, 2018; DOI: 10.3892/or.2018.6570

Following the publication of the above article, an interested reader drew to the authors' attention that Figs. 3C and E in the paper appeared to contain instances of duplicated data. The authors were able to consult their original data files, and realized that these figures had indeed been assembled incorrectly; subsequently, they requested that a corrigendum be published to take account of the errors that were made during the compilation of these figures.

Having investigated this matter in the Editorial Office, however, additional panels of overlapping data were identified, comparing between Figs. 3 and 5; specifically, overlapping data panels were also identified in panels in Figs. 3C, E and F, and 5C and D. The Editor of *Oncology Reports* has considered the authors' request to publish a corrigendum, but has decided to decline this request on account of the large number of errors that have been identified; rather, the article is to be be retracted from the Journal on the basis of an overall lack of confidence in the presented data. The Editor apologizes to the readership of the Journal for any inconvenience caused.

